# Proteomic profiling of HTLV-1 infected T-cells for the identification of potential biomarkers and therapeutic targets for HTLV-1 associated myelopathy/ tropical spastic paraparesis and adult T-cell leukemia

**DOI:** 10.1186/1742-4690-8-S1-A250

**Published:** 2011-06-06

**Authors:** Koji Ueda, Makoto Ishihara, Ayako Osawa, Naomi Senkoji, Natsumi Araya, Tomoo Sato, Atae Utsunomiya, Yoshihisa Yamano, Yusuke Nakamura, Hidewaki Nakagawa

**Affiliations:** 1 Laboratory for Biomarker Development, Center of Genomic Medicine, RIKEN, Tokyo, 108-8639, Japan; 2 Department of Molecular Medical Science, Institute of Medical Science, St. Marianna University School of Medicine, Kawasaki, 216-8512, Japan; 3 Department of Hematology, Imamura Bun-in Hospital, Kagoshima, 890-0064, Japan; 4 Laboratory of Molecular Medicine, Human Genome Center, Institute of Medical Science, the University of Tokyo, Tokyo, 108-8639, Japan

## 

Human T-lymphotropic virus type-1 (HTLV-1) can cause the development of HTLV-1 associated myelopathy/ tropical spastic paraparesis (HAM/TSP) and adult T-cell leukemia (ATL). These nervous system inflammation and cancer occur only in a very small proportion of infected individuals, indicating that several host and viral factors might associate with the risk of HAM/TSP or ATL.

The aim of this study is to identify a subset of proteins which would be applicable as the therapeutic targets or biomarkers for HAM/TSP or ATL. For this purpose, we employed comprehensive quantitative proteomics technologies. Peripheral blood mononuclear cells (PBMC) were collected from 6 uninfected volunteers, 4 asymptomatic carriers (AC), 10 HAM/TSP patients, and 9 ATL patients, followed by the specific selection of CD4+CD25+CCR4+ T cells, since this cell population was known as the predominant viral reservoir. The sorted T cells from 29 individuals were lysed, digested, and separately subjected to LC/MS/MS analyses. We integrated the 29 LC/MS/MS data into the Expressionist proteome database server platform and conducted label-free quantification analysis. The raw mass spectrometric data sets were processed and quantified on the RefinerMS module and statistical analysis was performed on the Analyst module. Eventually the results of ANOVA and the leave-one-out cross validation test revealed that the 100 peptide-panel allowed the clear classification of four pathological groups. In conclusion, our proteome profiling of CD4+CD25+CCR4+ T cells enabled us to identify the potential therapeutic or biomarker targets for HAM/TSP and/or ATL, while further validation studies with larger number of samples should be necessary.

